# Characterization of the *ompL1 *gene of pathogenic *Leptospira species *in China and cross-immunogenicity of the OmpL1 protein

**DOI:** 10.1186/1471-2180-8-223

**Published:** 2008-12-17

**Authors:** Haiyan Dong, Ye Hu, Feng Xue, Dexter Sun, David M Ojcius, Yafei Mao, Jie Yan

**Affiliations:** 1 Division of Basic Medical Microbiology, State Key Laboratory for Diagnosis and Treatment of Infectious Diseases, the First Affiliated Hospital of Medical College, Zhejiang University, Hangzhou Zhejiang, PR China; 2 Department of Medical Microbiology and Parasitology, Medical College of Zhejiang University, Hangzhou Zhejiang, PR China; 3 Medical School of Jinhua Professional Technique College, Jinhua Zhejiang, PR China; 4 New York Presbyterian Hospital & Hospital for Special Surgery, Weill Medical College, Cornell University, New York, NY, USA; 5 School of Natural Sciences, University of California, Merced, CA, USA

## Abstract

**Background:**

The usefulness of available vaccine and serological tests for leptospirosis is limited by the low cross-reactivity of antigens from numerous serovars of pathogenic *Leptospira *spp. Identification of genus-specific protein antigens (GP-Ag) of *Leptospira *would be important for development of universal vaccines and serodiagnostic methods. OmpL1, a transmembrane porin of pathogenic leptospires, was identified as a possible GP-Ag, but its sequence diversity and immune cross-reactivity among different serovars of pathogenic leptospires remains largely unknown.

**Results:**

PCR analysis demonstrated that the *ompL1 *gene existed in all 15 official Chinese standard strains as well as 163 clinical strains of pathogenic leptospires isolated in China. In the standard strains, the *ompL1 *gene could be divided into three groups (*ompL1/1*, *ompL1/2 *and *ompL1/3*) according to their sequence identities. Immune electron microscopy demonstrated that all products of the different gene types of *ompL1 *are located on the surface of leptospires. The microscopic agglutination test revealed extensive yet distinct cross-immunoagglutination among the antisera against recombinant OmpL1 (rOmpL1) and leptospiral strains belonging to different *ompL1 *gene types. These cross-immunoreactions were further verified by ELISAs using the OmpL1 proteins as the coated antigens in serum samples from 385 leptospirosis patients. All the antisera against rOmpL1 proteins could inhibit *L. interrogans *strain Lai from adhering to J774A.1 cells. Furthermore, immunization of guinea pigs with each of the rOmpL1 proteins could cause cross-immunoprotection against lethal challenge with leptospires from different *ompL1 *gene types.

**Conclusion:**

Three types of the *ompL1 *gene are present in pathogenic leptospires in China. OmpL1 is an immunoprotective GP-Ag which should be considered in the design of new universal vaccines and serodiagnostic methods against leptospirosis.

## Background

Leptospirosis, caused by infection with pathogenic *Leptospira *species belonging to different serogroups and serovars, is one of the most prevalent zoonotic diseases in the world [1-3]. A wide variety of serogroups and serovars have been identified along with endemicity which varies from region to region [4-6]. The leptospiral vaccines used currently are mainly multivalent dead whole-cell mixtures made of several local dominant serovars in different countries and regions. These vaccines, however, do not confer cross-protective immunity to the serogroups that are not represented in the vaccine [7,8], allowing the unrepresented serovars to continue causing outbreaks of leptospirosis. For instance, in a central province, Anhui, and an eastern province, Zhejiang, of China, *L. interrogans *serogroup Sejroe serovar Medanensis caused local outbreaks of leptospirosis [9-12]. In addition, vaccination with the whole-cell vaccines may lead to incomplete, short-term immunity as well as serious side effects [13-15]. A universal vaccine against leptospirosis is not available yet, making the identification of genus-specific protein antigens (GP-Ag) that display extensive cross-immunity very valuable for developing new vaccines and serodiagnostic methods.

Outer membrane proteins (Omps) are important pathogenic components and highly conserved in different serogroups and serovars of pathogenic leptospires. OmpL1, a transmembrane Omp with 320 amino acid residues, first reported by Haake and his colleagues in 1993, is a porin expressed by all the tested pathogenic *Leptospira *species [16-19]. However, the diversity of *ompL1 *gene sequences from different pathogenic *Leptospira *spp. and the distribution of the *ompL1 *gene in clinical isolates had not been characterized until now. Moreover, the cross-immunogenicity and immunoprotective effects of OmpL1 were mostly unknown.

In this study, we sequenced and analyzed *ompL1 *genes cloned from standard pathogenic strains of leptospires prevalent in China. Several prokaryotic recombinant products of the gene (rOmpL1) were expressed and their rabbit antisera were prepared. rOmpL1-based ELISAs were established to examine specific antibodies in sera from leptospirosis patients. In parallel, the microscopic agglutination test (MAT) was performed to detect the cross-immunoagglutination of different antisera against rOmpL1 proteins, and immunoelectron microscopy (IEM) was employed to localize OmpL1 on the leptospires. Finally, immunoprotection of rOmpL1 was tested in guinea pigs. Taken together, our results suggest that OmpL1 could be used as a major component of a universal and efficient immunogen for vaccination and also for diagnosis of leptospirosis.

## Results

### Amplification of *ompL1 *genes in the standard and clinical strains

All the 15 standard strains and 163 isolates of pathogenic *Leptospira *species (Table 1) carried the *ompL1 *gene, since amplicons with the expected size were produced by PCR from all the strains and isolates (data no shown).

**Table 1:** Background information on the leptospiral strains and serum specimens

Strain/Sera	Serovar	Serogroup	Genospecies
Standard strains (n = 15)			
Lai	Lai	Icterohaemorrhagiae	*L. interrogans*
Lin	Canicola	Canicola	*L. interrogans*
Tian	Pyrogenes	Pyrogenes	*L. interrogans*
Lin 4	Autumnalis	Autumnalis	*L. interrogans*
65-9	Australis	Australis	*L. interrogans*
Luo	Pomona	Pomona	*L. interrogans*
Lin 6	Grippotyphosa	Grippotyphosa	*L. interrogans*
P 7	Hebdomadis	Hebdomadis	*L. interrogans*
L 37	Paidjan	Bataviae	*L. interrogans*
L 183	Wolffi	Sejroe	*L. interrogans*
M 10	Javanica	Javanica	*L. borgpetersenii*
Pishu	Ballum	Ballum	*L. borgpetersenii*
55-52	Tarassovi	Tarassovi	*L. borgpetersenii*
Nan 10	Mini	Mini	*L. borgpetersenii*
L 105	Manhao 2	Manhao	*L. weilii*
Isolates (n = 163)			
85	Lai	Icterohaemorrhagiae	*L. interrogans*
5	Canicola	Canicola	*L. interrogans*
4	Autumnalis	Autumnalis	*L. interrogans*
18	Pomona	Pomona	*L. interrogans*
11	Grippotyphosa	Grippotyphosa	*L. interrogans*
15	Hebdomadis	Hebdomadis	*L. interrogans*
20	Medanesis	Sejroe	*L. interrogans*
5	Australis	Australis	*L. interrogans*
Patient sera (n = 385)			
191	Lai	Icterohaemorrhagia	*L. interrogans*
11	Canicola	Canicola	*L. interrogans*
13	Autumnalis	Autumnalis	*L. interrogans*
37	Pomona	Pomona	*L. noguchii*
31	Grippotyphosa	Grippotyphosa	*L. interrogans*
44	Hebdomadis	Hebdomadis	*L. interrogans*
45	Medanesis	Sejroe	*L. interrogans*
13	Australis	Australis	*L. interrogans*
Normal sera (n = 36)	/	/	/

### Molecular phylogeny and gene-typing of *ompL1 *gene

**Figure 1:**
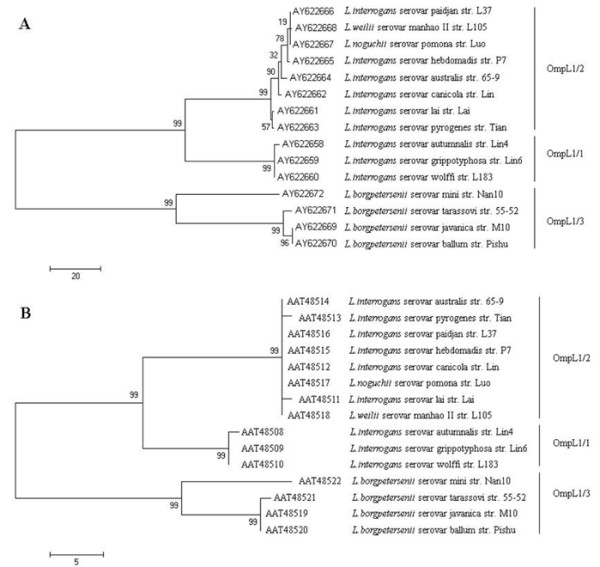
**Maximum parsimony trees for *ompL1 *nucleotide sequences (A) and its amino acid sequences (B) of 15 standard strains**. This figure shows the upper quartile, for the full image please see Additional file 1. The numbers at each fork node indicate the bootstrapping values (shown only when > 50%). RefSeq accession number and abbreviation for the organism are shown at each relevant branch. The scale bar indicates the number of character substitutions.

Based on the molecular phylogenetic relationship of their nucleotide sequences (GenBank Accession No.: AY622658–AY622672) and putative amino acid sequences (Figure 1, see Additional file 1 for details), o*mpL1 *genes from the pathogenic leptospires could be classified into three groups: *ompL1/1*, *ompL1/2 *and *ompL1/3 *(Table 2). Sequence identity of the putative amino acid sequences between *ompL1/1 *and *ompL1/2*, *ompL1/1 *and *ompL1/3*, and *ompL1/2 *and *ompL1/3 *gene types was 92.50%–93.44%, 85.31%–86.88%, and 85.31%–86.25%, respectively. In addition, the *ompL1 *gene sequencing data of 39 clinical strains also supported the classification into three groups (Table 2). Both the nucleotide and putative amino acid sequence identities among the clinical strains with the same *ompL1 *gene type were above 98% (data not shown).

**Table 2:** *ompL1 *gene types of standard strains and isolates of Leptospires

Species	Serogroup	Serovar	Strain (n)	*ompL1 *gene type		
				
				1	2	3
*L. interrogans*	Icterohaemorrhagiae	Lai	Lai		√	
			Wild (5)		√	
*L. interrogans*	Canicola	Canicola	Lin		√	
			Wild (5)		√	
*L. interrogans*	Australis	Australis	65-9		√	
			Wild (5)		√	
*L. interrogans*	Pomona	Pomona	Luo		√	
			Wild (5)		√	
*L. interrogans*	Hebdomadis	Hebdomadis	P 7		√	
			Wild (5)		√	
*L. interrogans*	Autumnalis	Autumnalis	Lin 4	√		
			Wild (4)	√		
*L. interrogans*	Grippotyphosa	Grippotyphosa	Lin 6	√		
			Wild (5)	√		
*L. interrogans*	Sejroe	Wolffi	L 183	√		
		Medanesis	Wild (5)	√		
*L. interrogans*	Bataviae	Paidjan	L 37		√	
*L. interrogans*	Pyrogenes	Pyrogenes	Tian			√
*L. borgpetersenii*	Javanica	Javanica	M 10			√
*L. borgpetersenii*	Ballum	Ballum	Pishu			√
*L. borgpetersenii*	Tarassovi	Tarassovi	55-52			√
*L. borgpetersenii*	Mini	Mini	Nan 10			√
*L. weilii*	Manhao	Manhao II	L 105		√	

### Data of secondary structure analysis of OmpL1 proteins

**Figure 2:**
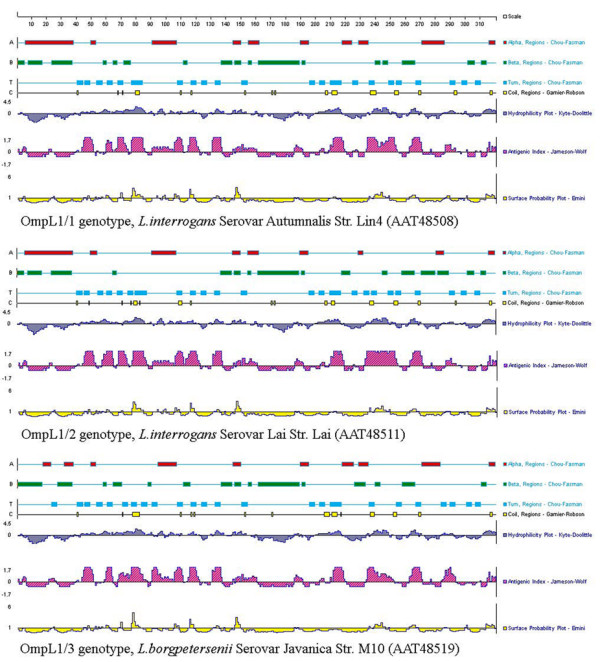
**Comparison of the predicted secondary structures and antigenic index of OmpL1 proteins**. This figure shows the upper quartile, for the full image please see Additional file 2. The related strains of the same gene type just show the same predicted structure topology. Thus, only the results from three representative strains are showed here.

All the three gene types of OmpL1 proteins had very similar predicted secondary structures and antigenic indexes (Figure 2, see Additional file 2 for details). The OmpL1 proteins had five main putative surface-membrane regions (70–80, 115–120, 145–150, 235–245, 305–310 amino acid residuals) and seven transmembrane regions (10–40, 120–130, 160–190, 215–225, 240–250, 270–280, and 295–305 amino acid residues). The only salient different was the presence of less alpha-helix in the N-terminal and beta-sheet in the C-terminal region of the OmpL1/3 sequence, compared to the sequences of OmpL1/1 and OmpL1/2.

### Identification of expressed rOmpL1 proteins and titers of rabbit antisera

**Figure 3:**
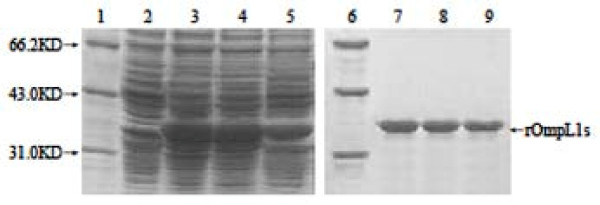
**Expression and purification of recombinant OmpL1 proteins**. Lanes 1 and 6: protein markers (BioColor); lane 2: pET42a with no inserted *ompL1 *genes; lanes 3 to 5: the expressed rOmpL1/1, rOmpL1/2 and rOmpL1/3, respectively; lanes 7 to 9: the purified rOmpL1/1, rOmpL1/2 and rOmpL1/3, respectively.

**Figure 4:**
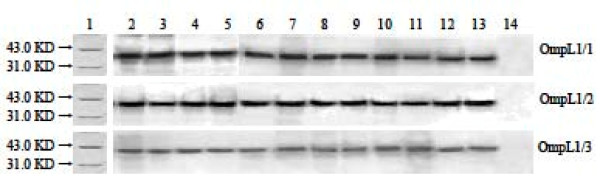
**Recognition of purified rOmpL1 proteins by sera from leptospirosis patients**. Lane 1: protein markers (BioColor); lanes 2–13: the rOmpL1/1, rOmpL1/2 and rOmpL1/3 hybridizing with the antisera from leptospirosis patients; lane 13: normal human serum used as control.

rOmpL1/1, rOmpL1/2 and rOmpL1/3 were well expressed after induction with IPTG and showed single bands in gel after purification by Ni-NTA-chromatography (Figure 3). Western blot analysis also indicated that serum samples from leptospirosis patients could recognize rOmpL1 proteins (Figure 4), implying that the proteins are immunogenic during natural infection. Immunodiffusion titers of all the rabbit antisera against rOmpL1 proteins were at least 1:4.

### Localization of OmpL1 proteins on leptospires

**Figure 5:**
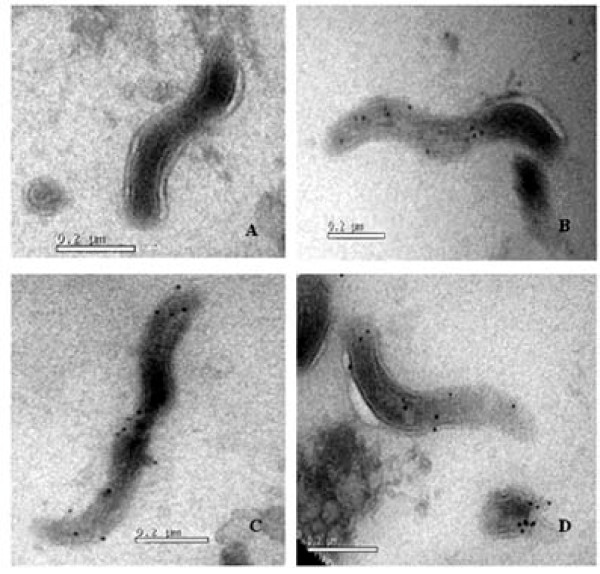
**The localization of OmpL1 on the surface of leptospires**. A: negative control; B to D: the immunogold particles binding to OmpL1/1, OmpL1/2 and OmpL1/3 on the surface of *L. interrogans *serovar Autumnalis strain Lin 4, serovar Lai strain Lai, and *L. borgpetersenii *serovars Ballum strain Pishu, respectively.

Using anti-rOmpL1/1, anti-rOmpL1/2 and anti-rOmpL1/3 sera as the primary antibodies, all the colloidal gold particles conjugated to secondary antibodies were bound on the surface of *L. interrogans *serovar Autumnalis strain Lin 4 (*ompL1/1 *gene type), serovar Lai strain Lai (*ompL1/2 *gene type), and *L. borgpetersenii *serovar Ballum strain Pishu (*ompL1/3 *gene type) (Figure 5).

### Titers of MAT

The antisera against rOmpL1/1, rOmpL1/2 and rOmpL1/3 were able to cross-agglutinate with MAT titers of 1:100 to 1:800 for all the 15 standard strains and 163 clinical strains of pathogenic leptospires (Table 3).

**Table 3:** MAT titers among anti-rOmpL1 antisera and 178 leptospiral strains

Strain	Serovar	Gene type	MAT titer (1:xxx)		
			
			Anti-OmpL1/1	Anti-OmpL1/2	Anti-OmpL1/3
Standards (n = 15)					
Lai	Lai	2	400	400	200
M 10	Javanica	3	100	100	400
Lin	Canicola	2	200	400	100
Pishu	Ballum	3	100	200	400
Tian	Pyrogenes	3	100	100	400
Lin 4	Autumnalis	1	400	400	200
65-9	Australis	2	200	400	100
Luo	Pomona	2	200	400	100
Lin 6	Grippotyphosa	1	400	400	200
P 7	Hebdomadis	2	200	400	100
L 37	Paidjan	2	200	400	100
55-52	Tarassovi	3	100	200	400
L 105	Manhao II	2	200	400	100
L 183	Wolffi	1	400	200	100
Nan 10	Mini	3	100	100	200
Isolates (n = 163)					
85	Lai		200~400	200~800	100~200
5	Canicola		200	400	100
4	Autumnalis		400	200~400	100
5	Australis		200	400	100
18	Pomona		200~400	200~800	100~200
11	Grippotyphosa		400~800	200~400	100~200
15	Hebdomadis		200~400	200~800	100~200
20	Medanesis		400	200~400	100~200

### Detection of antibodies against rOmpL1 proteins in leptospirosis patients

In ELISA assays using rOmpL1/1, rOmpL1/2 and rOmpL1/3, the positive rates for IgG in specimens from 385 leptospirosis patients using 1:100 and 1:200 serum dilutions were 87.8% (338/385) and 78.2% (301/385), 95.1% (366/385) and 82.1% (316/385), and 77.7% (299/385) and 65.5% (252/385), respectively (Table 4). When using 1:50 and 1:100 serum dilutions from the same specimens, the positive rates for IgM were 83.4% (321/385) and 72.7% (280/385), 87.0% (335/385) and 77.4% (298/385), and 74.3% (286/385) and 63.1% (243/385), respectively (Table 4).

**Table 4:** ELISA detection of rOmpL1-IgG/IgM in sera of leptospirosis patients

Sera	Cases (n)	Dilution (1:xxx)	IgG-ELISA positive (n)			IgM-ELISA positive (n)		
				
			rOmpL1/1	rOmpL1/2	rOmpL1/3	rOMPL1/1	rOMPL1/2	rOMPL1/3
Patients infected with:								
Icterohaemorrhagia	191	50	/	/	/	145	161	139
		100	159	187	149	132	152	128
		200	147	162	135	/	/	/
Canicola	11	50	/	/	/	9	11	9
		100	10	11	9	8	9	6
		200	8	10	6	/	/	/
Autumnalis	13	50	/	/	/	13	11	9
		100	13	12	10	10	8	7
		200	12	9	7	/	/	/
Pomona	44	50	/	/	/	38	41	31
		100	38	43	33	31	35	26
		200	33	37	27	/	/	/
Australis	13	50	/	/	/	12	13	10
		100	12	13	10	10	11	7
		200	10	11	8	/	/	/
Grippotyphosa	31	50	/	/	/	29	26	22
		100	30	26	23	23	21	16
		200	26	23	16	/	/	/
Hebdomadis	37	50	/	/	/	34	35	30
		100	33	36	30	29	30	25
		200	28	31	24	/	/	/
Sejroe	45	50	/	/	/	41	37	36
		100	43	38	35	37	32	28
		200	37	33	29	/	/	/
Total	385	50	/	/	/	321	335	286
		100	338	366	299	280	298	243
		200	301	316	252	/	/	/
Healthy individuals:	36	50	/	/	/	0.20 ± 0.06	0.20 ± 0.07	0.19 ± 0.07
		Cut-off	/	/	/	0.38	0.41	0.40
		100	0.17 ± 0.05	0.18 ± 0.05	0.17 ± 0.06	0.18 ± 0.05	0.17 ± 0.06	0.17 ± 0.05
		Cut-off	0.32	0.33	0.35	0.33	0.35	0.32
		200	0.14 ± 0.04	0.13 ± 0.05	0.15 ± 0.04	/	/	/
		Cut-off	0.26	0.28	0.27	/	/	/

Note: the mean at OD_490 _+ 3 SD = cut-off value

### Inhibition of adherence by antisera against rOmpL1 proteins

**Figure 6:**
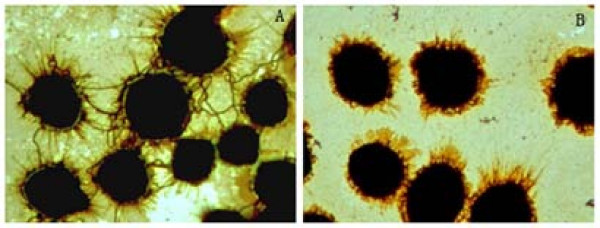
**Inhibition of leptospiral adherence to J744A.1 macrophages in the presence anti-rOmpL1 antiserum**. A: Leptospires binding to macrophages in the presence of irrelevant antisera. B: inhibition of leptospiral binding to macrophages in the presence of antiserum against recombinant OmpL1 protein (amplification ×1000).

Anti-rOmpL1/1 (1:200 dilution), anti-rOmpL1/2 (1:300 dilution) or anti-rOmpL1/3 (1:50 dilution) sera could completely inhibit the adherence of *L. interrogans *serovar Lai strain Lai to macrophages (Figure 6), demonstrating cross-inhibition effects among the different anti-sera.

### Immunoprotection due to immunization with rOmpL1 proteins

Immunization of guinea pigs with rOmpL1/1, rOmpL1/2 and rOmpL1/3 conferred a significant level of resistance against lethal challenge with pathogenic leptospires from the three different *ompL1 *groups (Table 5). However, the immunoprotective rates against the same *ompL1 *gene type leptospiral infection as the immunizing recombinant protein (62.5%–87.5%) were higher than when strains and proteins from different *ompL1 *gene types were used (25.0%–62.5%).

**Table 5:** Immunoprotective effects of rOmpL1 proteins in guinea pigs

Group	Animals (n)	Immunizing dosage (mg/kg)			Infecting Leptospiral	Survival/Death	Protective rate
					
		rOmpL1/1	rOmpL1/2	rOmpL/3	strain	(n/n)	(%)
A1	8	1.0	/	/	Lai	3/5	37.5
A2	8	2.0	/	/	Lai	5/3	62.5
A3	8	1.0	/	/	Lin 4	5/3	62.5
A4	8	2.0	/	/	Lin 4	7/1	87.5
A5	8	1.0	/	/	Pishu	2/6	25.0
A6	8	2.0	/	/	Pishu	3/5	37.5
B1	8	/	1.0	/	Lai	5/3	62.5
B2	8	/	2.0	/	Lai	6/2	75.0
B3	8	/	1.0	/	Lin 4	4/4	50.0
B4	8	/	2.0	/	Lin 4	5/3	62.5
B5	8	/	1.0	/	Pishu	2/6	25.0
B6	8	/	2.0	/	Pishu	3/5	37.5
C1	8	/	/	1.0	Lai	2/6	25.0
C2	8	/	/	2.0	Lai	3/5	37.5
C3	8	/	/	1.0	Lin 4	3/5	37.5
C4	8	/	/	2.0	Lin 4	4/4	50.0
C5	8	/	/	1.0	Pishu	5/3	62.5
C6	8	/	/	2.0	Pishu	6/2	75.0
D1	8	/	/	/	Lai	0/8	/
D2	8	/	/	/	Lin 4	0/8	/
D3	8	/	/	/	Pishu	0/8	/

## Discussion

Outer membrane proteins exposed on the surface of leptospires are known to react with the host cell and environment. Interestingly, lipopolysaccharide fractions confer protective immunity against challenge with homologous but not heterogonous leptospires, whereas protein extract induced significant protection against both types of challenge [20]. Thus far, a number of outer membrane proteins of leptospires, such as OmpL1, LipL32, LipL36, LipL41, LigA and LigB, have been cloned and characterized, and some of them have been shown to stimulate specific immunity in animal models [21-24]. Among all the leptospiral Omps, OmpL1 is a unique transmembrane protein that was confirmed to function as a porin, contribute to the survival of leptospires, and display synergetic immunoprotection with LipL41 [16-18]. However, major questions such as the distribution of *ompL1 *gene types in leptospiral strains, the exact localization of OmpL1, and cross-immunogenicity and immunoprotective effects of OmpL1 proteins remain unaddressed.

This study reveals that the *ompL1 *gene is present in the genomes of all the pathogenic leptospires tested. According to our alignment and phylogenetic analysis from the 15 standard strains of pathogenic *Leptospira *spp., three groups of *ompL1 *(*ompL1/1*, *ompL1/2 and ompl/3*) exist. However, the predicted secondary structure of the OmpL1 proteins revealed that there is little difference among the three groups. Thus, the differences in nucleotide sequences in the *ompL1 *gene types may not affect the immunogenicity and OmpL1 proteins, identifying OmpL1 as a genus-specific protein antigen.

Surface exposure is a key characteristic for an effective antigen. Although OmpL1 may be an outer membrane protein according to previous reports, the precise localization of OmpL1 still remained unclear. Leptospires possess both inner and outer membranes, but only the proteins expressed in the outer membrane are capable of interacting with the host immune system. To begin to characterize the localization, we used the prokaryotic recombinant expression technique to obtain a large amount of homogeneous OmpL1 proteins for preparation of immunoresponsive antisera from rabbits. Visualization by immuno-electron microscopy using anti-OmpL1 anti-sera confirmed that OmpL1 is located at the surface of the outer membrane of leptospires. MAT is a standard method for serodiagnosis of leptospirosis and serological classification of leptospires, for which live leptospiral cells are typically used [23]. In this study, we used MAT to examine cross-immunoagglutination among the antisera from rOmpL1 proteins and a large number of strains belonging to different pathogenic leptospiral serogroups/serovars. The results indicated that there is extensive cross-immunoagglutination between the different *ompL1 *gene types of pathogenic leptospiral strains and the OmpL1 antisera, and not surprisingly, the highest agglutination was observed between antisera from the same *ompL1 *gene types as the leptospiral strains. Furthermore, ELISA results revealed that OmpL1s-specific antibodies are produced in all the sera from leptospirosis patients, and the trends in cross-immunoreactivities in the different ELISA tests are also similar to those of MAT.

At least 75 serovars of pathogenic leptospires belonging to 18 serogroups have been found to date in China, but only a few of them frequently cause leptospirosis. According to the annual reports on leptospirosis from Chinese CDCs, *L. interrogans *serogroup Icterohaemorrhagiae serovar Lai is the most dominant pathogenic leptospires, responsible for approximately 75% of the morbidity in the country. The other pathogenic leptospires causing leptospirosis are the Grippotyphosa, Autumnalis, Australis, Pomona and Hebdomadis serovars [3,11,25]. Our study showed that all the 163 clinical strains of different pathogenic Leptospiral serogroup/serovars belong to either the *ompL1/1 *or *ompL1/2 *group (data not shown). Thus, we conclude that leptospires from the *ompL1/1 *and *ompL1/2 *groups are the most prevalent strains in China.

In this study, immunization with either rOmpL1/1, rOmpL1/2 or rOmpL1/3 proteins could enhance significantly survival of guinea pigs lethally challenged with pathogenic *Leptospira *species (Table 5). The immunoprotective rates in the groups, in which animals received a rOmpL1 for immunization and challenge with the same *ompL1 *gene type, appeared to be higher than those being challenged with different *ompL1 *gene types.

In a previous study [35], we found *L. interrogans *serovar Lai strain Lai could adhere to macrophages, and we here show that adherence to macrophages could be blocked with antisera against any of the rOmpL1 proteins, confirming cross-immunogenicity of OmpL1 proteins from the three different groups. All these data collectively suggest that OmpL1 is likely a genus-specific antigen.

## Conclusion

OmpL1 is a transmembrane protein extensively expressed in pathogenic leptospires. Although the *ompL1 *gene can belong to any of three different gene types, the OmpL1 proteins expressed by different gene types have conserved immunogenicity and the specific antibodies generally exist in sera of leptospirosis patients. OmpL1 should thus be viewed as a potential candidate of genus-specific antigen for the development of new universal vaccines and serodiagnostic methods for leptospirosis.

## Methods

### Leptospiral strains and serum specimens

The National Institute for the Control of Pharmaceutical and Biological Products (NICPBP) in Beijing, China, provided all 15 official Chinese standard strains belonging to fifteen different pathogenic serogroups of *Leptospira*. In addition, 163 clinical pathogenic Leptospiral isolates and 385 serum specimens from leptospirosis patients with MAT titers of ≥ 1:100 were provided by the Centers of Disease Prevention and Control of Sichuan, Anhui and Zhejiang provinces in China during 2004–2007 (Table 1). All the leptospiral strains were cultured in liquid Korthof medium containing 8% rabbit serum at 28°C [2,25]. All the patients were clinically diagnosed suffering from acute leptospirosis based on their medical history and clinical symptoms, including fevers, jaundice, hemorrhaging, myalgia and lymphadenectasis, as well as laboratory examination. Finally, 36 sera samples with negative results by MAT from healthy individuals for routine somatoscopy were offered by the First Affiliated Hospital of Zhejiang University. Informed written consent for sample collection was obtained from all participants and individuals with ethical approval from the Ethics Committee of Zhejiang University. This research was conducted in accordance with the Declaration of Helsinki and with the Guide for Care and Use of Laboratory Animals as adopted and promulgated by the United States National Institutes of Health. All experimental protocols were approved by the Ethics Committee of Zhejiang University.

### Cell line and cell culture

The murine monocyte/macrophage-like cell line, J774A.1, was provided by the Cell Bank of the Institute of Cell Biology in Shanghai, Chinese Academy of Science, and was maintained in RPMI 1640 medium (GiBco, Grand Island, USA) supplemented with 10% fetal bovine serum (FBS) (GiBco), and 100 U/ml penicillin and 100 μg/ml streptomycin. The cells were cultured in 5% CO_2 _at 37°C.

### Amplification and sequencing of *ompL1 *genes

Leptospiral DNA was extracted using Bacterial Genomic DNA Extraction Kit (BioColor BioScience & Technology Co., Shanghai, China) and then dissolved in TE buffer. Density and purity of the extracted DNAs were detected by UV spectrophotometery. One pair of primers was applied to amplify the entire *ompL1 *gene. The upstream primer was 5'-CCG CATATG (Nde I) ATC CGT AAC ATA AGT AAG-3' and the downstream primer was 5'-CCG CTCGAG (Xho I) GAG TTC GTG TTT ATA ACC-3'. A High Fidelity PCR Kit (TaKaRa, Dalian, China), in which Taq-Pfu mixture is used as DNA polymerase, was used to amplify the target gene. The total volume per PCR was 50 μl which included 20 pmol of each of the primers, 2.5 U Taq-Pfu DNA polymerase, and 100 ng DNA templates. The reaction mixture was initiated by incubation at 94°C for 5 min, followed by 30 cycles of amplification at 94°C for 30 s, 55°C for 30 s and 72°C for 90 s, and then incubation at 72°C for 10 min. The products were detected in 1.5% ethidium bromide pre-stained agarose gel by agarose electrophoresis. The target products were predicted to be 960 base pairs (bp) in size. To obtain more accurate sequence data, the PCR products were purified using PCR Products Purification Kit (BioColor) and then ligated into plasmid pMD 18-T using T-A Cloning Kit (TaKaRa) [26]. The cloned *ompL1 *genes of 15 standard strains and 39 clinical strains were sequenced by Invitrogen (Shanghai, China).

### Phylogenetic analysis and secondary structure prediction of OmpL1 protein

The similarity and homology of sequences were first evaluated with the BLASTN program of NCBI. Alignments were carried out using ClustalX 1.83 software [27] and manually adjusted using GeneDoc 2.7 software. The amino acid sequences were deduced from their nucleotide sequences. Phylogenetic analysis was carried out with Maximum Parsimony (MP) and Maximum Likelihood (ML) optimality criteria of the PAUP* package (Version 4.0b10) [28] and MEGA 3.1 software [29]. And the probability prediction for secondary structures [30,31], including antigenic analysis [32] of OmpL1 proteins, was computed using the protean program in DNAStar™ software package.

### Prokaryotic expression and preparation of antisera against OmpL1 proteins

The recombinant plasmids *pMD18-T-ompL1/1/2/3 *as well as vector pET42a (Novagen, USA) were respectively digested with Nde I and Xho I (TaKaRa). The recovered *ompL1 *segments were respectively linked with the linearized pET42a by T4 DNA ligase (TaKaRa) and then transformed into *E. coli *BL21DE3 (Novagen). The three constructed prokaryotic expression systems were induced with 0.5 mM IPTG in LB medium to express recombinant OmpL1 (rOmpL1). Expression of rOmpL1 was examined by SDS-PAGE plus Gel Image Analyzor (BioRad, USA). The expressed rOmpL1 proteins were extracted and purified by Ni-NTA affinity chromatography. New Zealand white Rabbits provided by the Laboratory Animal Center, Zhejiang University, were immunized intradermally with each of the purified rOmpL1 proteins pre-mixed with Freund's adjuvant for four times at an interval of once a week. On the 15th day after the last immunization, the rabbit sera were collected and the Immunodiffusion test was used to examine the titers of antisera [24].

### Western blot assay

Western blot analysis was used to identify the immunoreactivity of rOmpL1 proteins using 12 serum samples from the patients diluted at 1:1000 (the same dilution of normal human sera was used as control) as the primary antibodies, and 1:3000 diluted HRP-labeled goat anti-human IgG (Jackson ImmunoResearch Laboratories Inc., USA) as the secondary antibody.

### Immuno-electron microscopy

Freshly-cultured *L. interrogans *serovar Autumnalis strain Lin 4 (*ompL1/1 *gene type), serovar Lai strain Lai (*ompL1/2 *gene type), and *L. borgpetersenii *serovar Ballum strain Pishu (*ompL1/3 *gene type) were centrifuged at 12, 000 rpm for 15 min at 15°C. The precipitates were fixed with fresh 2% paraformaldehyde at 4°C for 24 h. Prepared sections were exposed to 5% FBS at room temperature for 30 min to block unspecific antigens, and then incubated with each 1:1000 diluted rOmpL1s rabbit antisera or preimmune normal rabbit sera (negative control) as the first antibody and 1:2000 diluted colloidal gold (12 nm)-labeled goat anti-rabbit IgG (Jackson ImmunoResearch Laboratories) as the second antibody. The locations of OmpL1s were determined under transmission electron microscope (Philips TECNAL-10, Eindhoven, Holland)[16].

### Microscopic agglutination test

The microscopic agglutination test (MAT) was used to determine cross-immunoagglutination under dark-field microscopy using rabbit antisera against the rOmpL1 proteins reacting with the 15 freshly-cultured standard strains and 163 clinical isolates of leptospires, in which normal saline was used as negative control [2,33].

### ELISA assay with rOmpL1 proteins

Enzyme-linked immunosorbent assays (ELISAs) were used to detect rOmpL1/1 or rOmpL1/2-specific IgM or IgG. Briefly, 96-well plates were coated with 50 μg/ml rOmpL1/1 or rOmpL1/2 (100 μl per well) at 4°C overnight, and then incubated with 5% FBS for 30 min to block unspecific binding. Using the diluted serum specimens from patients or normal individuals (1:50 or 1:100 dilution for IgM detection, and 1:100 or 1:200 dilution for IgG detection) as the primary antibody, HRP-labeling goat anti-human IgM (1:3000 dilution) or IgG (1:4000 dilution) (Jackson ImmunoResearch Laboratories) as the secondary antibody, and o-phenvlenediamine (OPD) as the substrate, the rOmpL1 proteins-specific IgM or IgG were examined under a Microplate Reader (Model 550, BioRad). OD_490 _values over the mean plus 3 SD of the 36 normal serum specimens were defined as positive [34].

### Adherence inhibition test

J774A.1 cells (1 × 10^5 ^cells in 1 ml medium per well) were inoculated in 12-well plates (Corning, USA) containing glass cover slips, incubated at 37°C for 24 h. Freshly-grown cultures of *L. interrogans *serovar Lai strain Lai were centrifuged at 10,000 rpm for 15 min at 15°C, and the precipitates were then suspended in antibiotics-free RPMI 1640 medium (GiBco) to a final concentration of 10^8^/ml. The antisera against rOmpL1 proteins were serially double-diluted with antibiotics-free RPMI 1640 medium (1:2 to 1:32), then mixed with the same volumes of each leptospiral suspension. The mixtures were incubated at 37°C for 1 h. The medium in the 12-well plates was removed, and then the plates were repeatedly washed with antibiotic-free RPMI1640 medium. The mixtures were added into the plates to incubate at 37°C for 1 h. After the incubation, the plates were repeatedly washed with PBS to remove non-adhered leptospires, and the Fontana silver-stained leptospires adhering J774A.1 cells were observed under light microscope [35]. In this test, preimmune rabbit serum instead of the antisera was used as control.

### Immunoprotective test in guinea pigs

Dunkin-Hartley guinea pigs (150 ± 5 g, 3 weeks old) used in this study were provided by Laboratory Animal Center of Zhejiang University (certificate no. SCXK [zhe]2007-0030). *L. interrogans *serovar Lai strain Lai, serovar Autumnalis strain Lin 4, and *L. borgpetersenii *serovar Bullum stain Pishu were maintained by serial passage in guinea pigs for preservation of virulence before use for challenge. Eighteen groups of guinea pigs (A1–6, B1–6, and C1–C6) were subcutaneously immunized twice at an interval of once a week with different dosages of rOmpL1/1, rOmpL1/2 and rOmpL1/3, respectively. In addition, another three groups of guinea pigs (D1-3) were immunized with bovine serum albumin (BSA) (Sigma, USA) as control. Each protein antigen was pre-mixed with 0.7 mg aluminum hydroxide (Sigma) [36]. On the 15th day after the last immunization, the animals were challenged intraperitoneally with lethal doses of strain Lai, strain Lin 4 or strain Pishu and then observed for another 7 days.

## Authors' contributions

HD carried out the molecular genetic studies, immunoassays, drafted the manuscript. YH: carried out the immunoelectron microscope assay. FX carried out the bioinformatics analysis and immunoprotective test. JM carried out the immunoassays, cultured the leptospires. DS participated in the design of the study, analysis and interpretation of data. DO participated in study design and coordination, revised the manuscript. JY conceived of the study, and participated in its design and coordination. All authors read and approved the final manuscript, agreed to be published.

## Supplementary Material

Additional file 1 **Full image of maximum parsimony trees for *ompL1 *nucleotide sequences (A) and its amino acid sequences (B) of 15 standard strains.** This figure showed the phylogenetic relationship of nucleotide sequence and amino acid sequences of Chinese 15 standard strains of pathogenic leptospires. Click here for file

Additional file 2 **Full image of comparison of the predicted secondary structures and antigenic index of OmpL1 proteins.** This figure showed the predicted secondary structures and antigenic index of OmpL1 proteins belongs to three gene types (*ompL1/1, ompL1/2, and ompL1/3*). Click here for file
