# Supplementary figures and images for: Characterization of the ompL1 gene of pathogenic Leptospira species in China and cross-immunogenicity of the OmpL1 protein
Source: BMC Microbiol. 2008 Dec 17;8:223. doi: 10.1186/1471-2180-8-223 (PMC2632671; doi:10.1186/1471-2180-8-223)

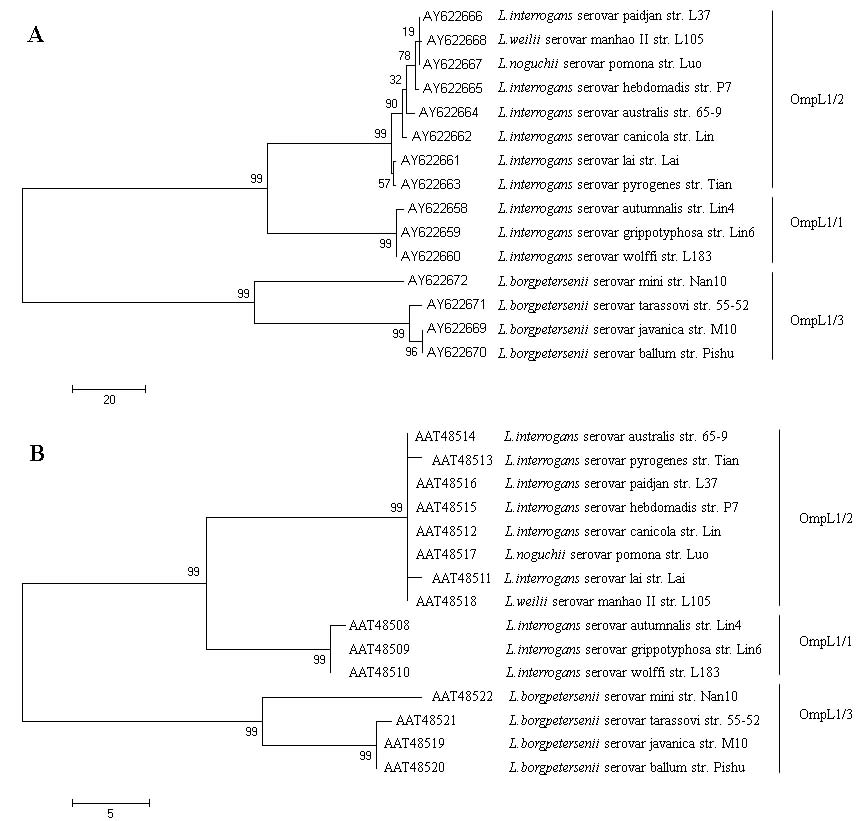

Supplement: Additional file 1 — Full image of maximum parsimony trees for ompL1 nucleotide sequences (A) and its amino acid sequences (B) of 15 standard strains. This figure showed the phylogenetic relationship of nucleotide sequence and amino acid sequences of Chinese 15 standard strains of pathogenic leptospires. [file 1471-2180-8-223-S1.tiff]

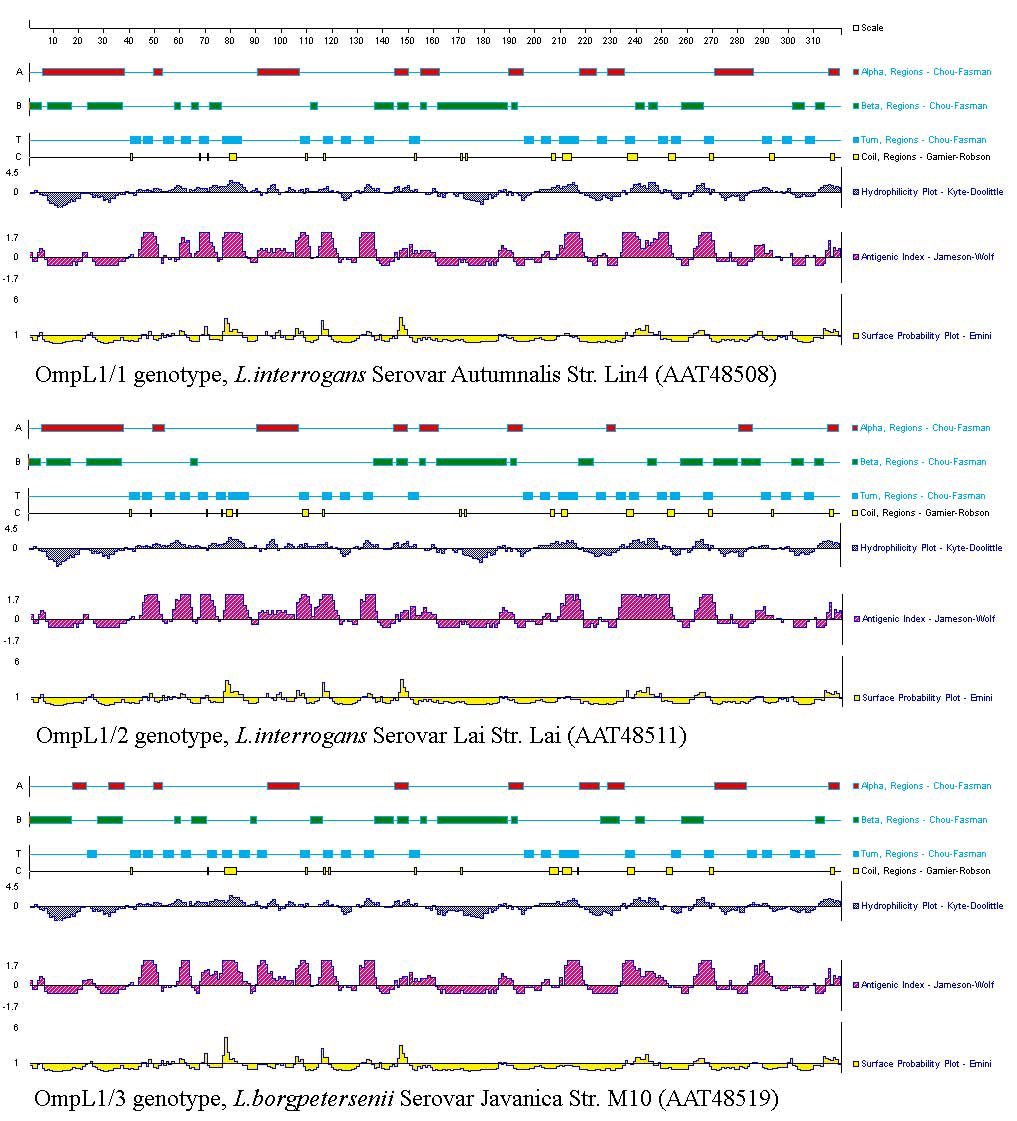

Supplement: Additional file 2 — Full image of comparison of the predicted secondary structures and antigenic index of OmpL1 proteins. This figure showed the predicted secondary structures and antigenic index of OmpL1 proteins belongs to three gene types (ompL1/1, ompL1/2, and ompL1/3). [file 1471-2180-8-223-S2.tiff]
